# Identification of Luteolin as Enterovirus 71 and Coxsackievirus A16 Inhibitors through Reporter Viruses and Cell Viability-Based Screening

**DOI:** 10.3390/v6072778

**Published:** 2014-07-17

**Authors:** Lin Xu, Weiheng Su, Jun Jin, Jiawen Chen, Xiaojun Li, Xuyuan Zhang, Meiyan Sun, Shiyang Sun, Peihu Fan, Dong An, Huafei Zhang, Xiguang Zhang, Wei Kong, Tonghui Ma, Chunlai Jiang

**Affiliations:** 1 School of Life Sciences, Jilin University, Changchun 130012, China; E-Mails: xulinjlu@hotmail.com (L.X.); suweiheng@163.com (W.S.); jun.jin@pasteur.fr (J.J.); JiawenChen229@gmail.com (J.C.); lixiaojun3345@163.com (X.L.); zxy_better@163.com (X.Z.); sunmy990@163.com (M.S.); sunbaoshiyang@163.com (S.S.); fph121@163.com (P.F.); andonginlab@sina.cn (D.A.); bszhfing@163.com (H.Z.); zhangxiguang@outlook.com (X.Z.); weikong@jlu.edu.cn (W.K.); 2 National Engineering Laboratory for AIDS Vaccine, Jilin University, Changchun 130012, China; 3 Key Laboratory for Molecular Enzymology and Engineering, The Ministry of Education, Jilin University, Changchun 130012, China; 4 College of Basic Medical Sciences, Dalian Medical University, Dalian 116000, China

**Keywords:** enterovirus 71, coxsackievirus A16, luteolin, reporter virus, high‑throughput assay, antiviral drug discovery

## Abstract

Hand, foot and mouth disease (HFMD) is a common pediatric illness mainly caused by infection with enterovirus 71 (EV71) and coxsackievirus A16 (CA16). The frequent HFMD outbreaks have become a serious public health problem. Currently, no vaccine or antiviral drug for EV71/CA16 infections has been approved. In this study, a two-step screening platform consisting of reporter virus-based assays and cell viability‑based assays was developed to identify potential inhibitors of EV71/CA16 infection. Two types of reporter viruses, a pseudovirus containing luciferase-encoding RNA replicons encapsidated by viral capsid proteins and a full-length reporter virus containing enhanced green fluorescent protein, were used for primary screening of 400 highly purified natural compounds. Thereafter, a cell viability-based secondary screen was performed for the identified hits to confirm their antiviral activities. Three compounds (luteolin, galangin, and quercetin) were identified, among which luteolin exhibited the most potent inhibition of viral infection. In the cell viability assay and plaque reduction assay, luteolin showed similar 50% effective concentration (EC_50_) values of about 10 μM. Luteolin targeted the post-attachment stage of EV71 and CA16 infection by inhibiting viral RNA replication. This study suggests that luteolin may serve as a lead compound to develop potent anti-EV71 and CA16 drugs.

## 1. Introduction

Hand, foot and mouth disease (HFMD) is a common infectious illness in infants and children. HFMD generally presents as a mild febrile disease with mild exanthems, but some patients develop severe neurological diseases, including aseptic meningitis, fatal encephalitis, and acute flaccid paralysis [1,2,3,4]. Data reported by World Health Organization showed that over two million cases were diagnosed in the year 2013, including China (1,855,457 cases), Japan (300,314 cases), Vietnam (71,627 cases), and Singapore (31,780 cases) [5].

Enterovirus 71 (EV71) and coxsackievirus 16 (CA16), both belonging to the *Picornaviridae* family, are the causative agents of HFMD [6]. EV71 infection can cause severe complications and mortality [7], while nearly 60% HFMD cases are caused by CA16 [8,9]. Importantly, the co‑circulation and recombination of EV71 and CA16 have been reported to appear in serious outbreaks in Malaysia, Mainland China, and Taiwan [10,11]. This makes the control of epidemic HFMD more complex and difficult.

Currently, there is no available specific vaccine or antiviral drug against EV71 and CA16 [12]. Three candidate vaccines against EV71 have recently completed Phase III trials in Mainland China, all of which have shown good safety and mediated protective effects [13]. Regarding drug discovery, previous studies have reported the anti-EV71/CA16 activities of several natural products (e.g., chrysosplenetin, pendulentin, matrine, glycyrrhizic acid) [14,15,16] and synthetic compounds (e.g., BPROZ series, DTriP‑22, rupintrivir) [17,18,19]. However, none of them has been advanced to human clinical trials.

The development of antiviral compounds requires appropriate screening assays, which should be rapid and reliable. The current commonly used antiviral assays are based on virus-induced cytopathic effects (CPE). These methods have disadvantages of being time-consuming and labor-intensive, which limit their use for high throughput screening (HTS). In some cases, pseudoviruses have been designed to contain reporter proteins and used for HTS platforms to discover viral infection inhibitors [20,21]. Nevertheless, these tools are unable to represent the entire replication cycle. These shortcomings can be avoided by employing viruses production from full-length infectious clones that contain convenient reporters, which have been generated for various RNA viruses including Visna virus [22], Chandipura virus [23], hepatitis C virus [24], coxsackievirus B3 [25] and EV71 [26], but not for CA16. Due to the lack of a CA16 high infective cell model, full-length CA16 infectious clones are often difficult to manipulate. Fortunately, this problem has been solved since we have established EV71 and CA16 susceptible cell lines, which stably overexpress hSCARB2 (human scavenger receptor class B, member 2), the receptor of EV71 and CA16 [27,28].

In this study, we established two reporter virus-based HTS assays as primary screens for EV71/CA16 inhibitors: (1) a luciferase reporter infection assay using a pseudovirus (luciferase‑encoding RNA replicons encapsidated by viral capsid proteins), which allows screening for inhibitors of viral infection; (2) an enhanced green fluorescent protein (EGFP) reporter infection assay using a full-length infectious clone, which allows screening for inhibitors of any step(s) of the replication cycle. These two assays were utilized for the first time to screen EV71/CA16 inhibitors from a natural compounds library. After the primary screening, a number of hits were re-evaluated by a cell viability-based secondary screening assay with wild-type viruses. Luteolin was selected for having the most potent inhibition of EV71/CA16 infection, and was further evaluated from various aspects such as 50% effective concentration (EC_50_), 50% cytotoxic concentration (CC_50_), 50% selectivity index (SI_50_) and addressed infectious stage. 

## 2. Materials and Methods

### 2.1. Cells and Drug Library

293T cells, RD cells (human embryonal rhabdomyosarcoma), and Vero cells were cultured as monolayers in Dulbecco’s modified Eagle medium (DMEM) (Sigma) supplemented with 10% fetal calf serum (FCS) (10% FCS-DMEM). The RD-SCARB2 (RDS) cell line stably overexpressing hSCARB2, which has been described previously [28], was cultured in 10% FCS-DMEM supplemented with puromycin (0.5 μg/mL; Clontech, Mountain View, CA, USA).

The drug library used in this study is a natural product library that contains 400 highly purified compounds (purchased from National Institutes for food and drug control, Beijing, China). All compounds in the library are highly purified and have known chemical structures with low molecular weight. These compounds were dissolved in dimethyl sulfoxide (DMSO) to 20 mM. The final compound concentration used in all screening assays was 100 μM, with a final DMSO concentration of 0.5%.

### 2.2. Viruses

#### 2.2.1. Wild-Type Viruses

EV71 (genotype C4b) was provided by the Chinese Center for Disease Control and Prevention. CA16 (Genbank accession no. JF695003.1) was provided by Henan Provincial Center for Disease Control and Prevention. Both viruses were grown in RDS cells.

#### 2.2.2. EV71/CA16-Luciferase Pseudoviruses

EV71 and CA16 pseudoviruses containing the firefly luciferase gene replacing the P1 gene were prepared as described previously [29,30].

#### 2.2.3. CA16-EGFP and EV71-EGFP Viruses

##### 2.2.3.1. Construction of Plasmids

The pT7-CA16-EGFP plasmid, which was commercially synthesized (GenBank accession no. AF177911.1, Generay Bio Co. Ltd., Shanghai, China), contains the reverse transcribed full-length CA16 genome with EGFP inserted in the frame between the 5'-NTR and VP4. The 2A protease cleavage site (ITTLG) was designed downstream the EGFP gene in order to release the reporter protein. The hepatitis delta virus (HDV) ribozyme (Rib) sequence was added at the 3'end, followed by the T7 terminator [31]. The pT7-EV71-EGFP plasmid (GenBank accession no. HM002485.1), which was constructed by the same strategy, was provided by Liguo Zhang at the Institute of Biophysics, Chinese Academy of Sciences (Beijing, China).

The plasmid pcDNA3.1-T7 RNA Pol, which expresses T7 RNA polymerase under control of CMV promoter, was constructed as described previously [29,30].

##### 2.2.3.2. Virus Production

293T cells were co-transfected with pcDNA3.1-T7 RNA Pol and pT7-EV71-EGFP or pT7-CA16-EGFP using Lipofectamine 2000 (Invitrogen, Carlsbad, CA, USA). EGFP reporter viruses were aliquoted and stored at −80 °C until use.

##### 2.2.3.3. Virus Titration

Infectious titers were obtained using RDS, RD, and Vero cells as previously described [32]. Cytopathic effects were visible after incubation for 96 h, and the 50% tissue culture infectious doses (TCID_50_)/mL were calculated by the Reed and Muench method [33].

##### 2.2.3.4. Reverse Transcription PCR (RT-PCR)

Viral RNA was extracted from virus infected supernatant using the QIAamp^®^ viral RNA mini kit (Qiagen, Hilden, Germany). Reverse transcription-polymerase chain reaction (RT-PCR) was performed using PrimeScript^®^ RT-PCR Kit (Takara Bio, Otsu, Japan) and primers located in the 5'NTR (5' TTAAAACAGCCTGTGGGTTG 3') and VP4 (5' GGTGGAGACTTGTGACCCCAT 3') region. 

### 2.3. Reporter Virus-Based Primary Screening

Two types of reporter virus-based HTS assays were used to screen antiviral drugs of the natural product library. The first was an EGFP-based antiviral assay. We screened the compounds with EGFP reporter viruses by modifying a previously described assay [34]. Briefly, RDS cells were cultivated in 96-well plates to 90% confluency, then the medium was aspirated followed by infection with 50 μL of CA16-EGFP or EV71-EGFP at the MOI (multiplicity of infection) of 5.56 and simultaneously treated with 50 μL of each compound solution diluted in 2% FCS-DMEM at a final concentration of 100 μM. Infected cells were incubated at 37 °C for 24 h. The EGFP fluorescence signal was measured using PerkinElmer VICTOR^TM^ X2 (Waltham, MA, USA) at 485 nm excitation and 535 nm emission wavelengths, respectively. 

The second type was the luciferase-based antiviral assay. Luciferase gene containing EV71 and CA16 pseudoviruses were developed into an antiviral screening assay by modifying the assay described previously [29,30]. RDS cells were cultivated in 96-well plates to 90% confluency, then the medium was aspirated followed by infection with 50 μL EV71 pseudoviruses (200 CCID_50_, 50% cell culture infective dose) or CA16 pseudoviruses (400 CCID_50_) and simultaneously treated with 50 μL of each compound solution diluted in 2% FCS-DMEM at a final concentration of 100 μM. Infected cells were incubated at 37 °C for 15 h, and the luminescence signal was measured using PerkinElmer VICTOR^TM^ X2 (Waltham, MA, USA).

### 2.4. Cell Viability-Based Secondary Screening

After the primary screening assay, the candidate compounds were further assessed by using a CellTiter-Glo® Luminescent Cell Viability Assay (Promega, Madison, WI, USA) to detect cell viability and virus-induced CPE as previously described [35]. Briefly, RDS cells were seeded in 96‑well plates to 90% confluency, and the medium was aspirated followed by infection with 50 μL EV71 or CA16 (MOI = 0.005) and simultaneously treated with 50 μL of each compound solution diluted in 2% FCS-DMEM at a final concentration of 100 μM. After incubation at 37 °C for 48 h, 100 μL of CellTiter-Glo® reagent was added to each well, and the luminescence signals were measured immediately using PerkinElmer VICTOR^TM^ X2 (Waltham, MA, USA). 

### 2.5. Determination of Antiviral EC_50_ and CC_50_ of Luteolin

Using the same CellTiter-Glo® Luminescent Cell Viability Assay, we also assessed cell viability with serially diluted luteolin (0.39–200 μM) to determine the CC_50_, defined as a 50% reduction in luminescence compared to control wells. Similarly, the EC_50_ was defined as the compound concentration that led to retention of 50% luminescence from infected cells. CC_50_ and EC_50_ were calculated using linear regression analysis [36].

### 2.6. Plaque Reduction Assay

RDS cells were seeded in 12-well plates to 90% confluency and infected with EV71 or CA16 at 100–150 pfu (plaque forming units) per well in the presence or absence of the serially diluted luteolin. After incubation at 37 °C for 1 h, the inoculum was replaced with a medium (2% FCS-DMEM) containing luteolin at the corresponding concentrations with 1% agarose and incubated at 37 °C for 72 h. After incubation, the cells were stained with 0.05% crystal violet. The concentration that reduced 50% plaque numbers was determined as EC_50_.

### 2.7. Viral Replication Assay

Confluent RDS cells in 24-well plates were inoculated with EV71 and CA16 (MOI = 1) at 4 °C for 1 h. Then the cells were washed and treated with or without luteolin (50 μM) and incubated at 37 °C. After different hours post-infection, the cells were washed, harvested, and frozen at −80 °C prior to real-time RT-PCR.

### 2.8. Real-Time RT-PCR of Viral RNA

Viral RNA was extracted using the QIAamp^®^ viral RNA mini kit (Qiagen). Real-time RT-PCR was carried out using the One Step SYBR^®^ PrimeScript™ RT-PCR Kit II (Takara Bio) and 5'NTR-specific primers as described [37].

## 3. Results

### 3.1. Construction and Characterization of EGFP Reporter Viruses

**Figure 1:**
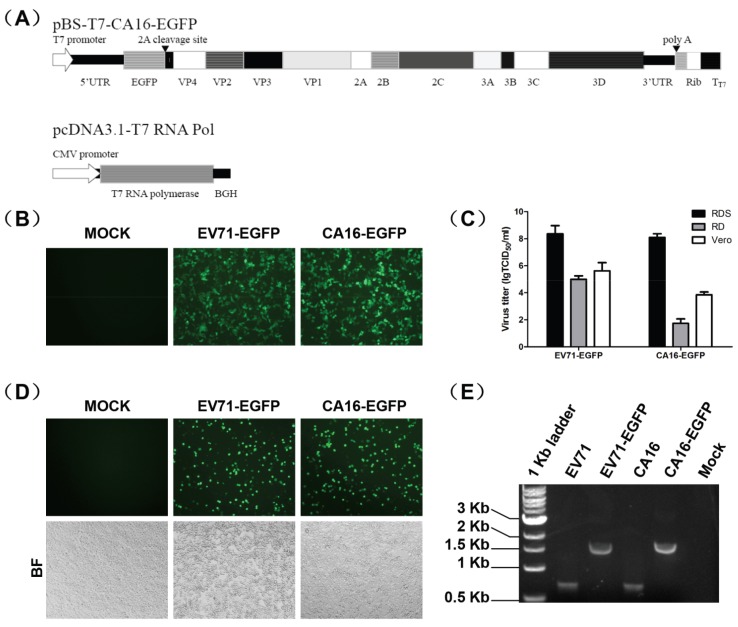
Construction and characterization of enhanced green fluorescent protein (EGFP) reporter viruses. (**A**) Schematic of the EGFP-CA16 reporter virus. (**B**) EGFP expressions in cells co-transfected with pcDNA3.1-T7 RNA Pol and pT7-EV71-EGFP or pT7-CA16-EGFP for 24 h. (**C**) Comparison of the susceptibility of recovered EGFP reporter viruses in different cell lines (RD-SCARB2 (RDS), human embryonal rhabdomyosarcoma (RD) and Vero). Data are presented as the mean ± SD of three independent experiments. (**D**) Detection of EGFP expression and cytopathic effects (CPE) of P5 reporter viruses in RDS cells at 24 hpi. (**E**) Detection of EGFP gene in culture supernatants of P5 reporter viruses by reverse transcription-polymerase chain reaction (RT-PCR).

To obtain EV71-EGFP and CA16-EGFP reporter viruses, 293T cells were co-transfected with pT7‑EV71-EGFP or pT7-CA16-EGFP and pcDNA3.1-T7 RNA Pol (Figure 1A). The expression of T7 RNA polymerase activated the transcription of plasmids with T7 promoter. EGFP expression and the reporter virus-induced CPE were observed under a fluorescence microscope at 24 h post‑transfection (hpt) (Figure 1B), indicating that the EV71-EGFP and CA16-EGFP reporter viruses were successfully recovered. 

Subsequently, we compared the susceptibility of different cell lines (RDS, RD and Vero) to the recovered EGFP reporter viruses by titration. As shown in Figure 1C, the virus titer of EV71‑EGFP titrated in RDS cells (~10^8^ TCID_50_/mL) was over 10^3^-fold higher than in RD cells (~10^5^ TCID_50_/mL) and Vero cells (~10^4^ TCID_50_/mL). For CA16-EGFP, the virus titer was dramatically elevated in RDS cells (~10^8^ TCID_50_/mL) and was over 10^4^-fold higher than in RD cells (~10^2^ TCID_50_/mL) and Vero cells (~10^4^ TCID_50_/mL). Thus, the RDS cell line was determined to be a suitable cell culture model for EGFP reporter viruses. 

The recovered EGFP reporter viruses from 293T cells (defined as passage 0, P0) were blind passaged for five rounds in RDS cells. Strong EGFP signals were detected in P5 virus-infected cells (Figure 1D), proving that the EGFP gene was stably maintained. The P5 viruses were further characterized by RT-PCR with wild-type viruses as positive controls. A specific band at 1.5 kb was amplified, which corresponded to the expected size for EV71-EGFP and CA16-EGFP and included the 5'NTR and EGFP gene. A 0.7 kb band was amplified in wild type viruses due to their lack of the EGFP gene (Figure 1E).

### 3.2. HTS Screening System to Identify Anti-EV71 and CA16 Drugs

#### 3.2.1. Primary Screening Using Reporter Virus-Based Methods

**Figure 2:**
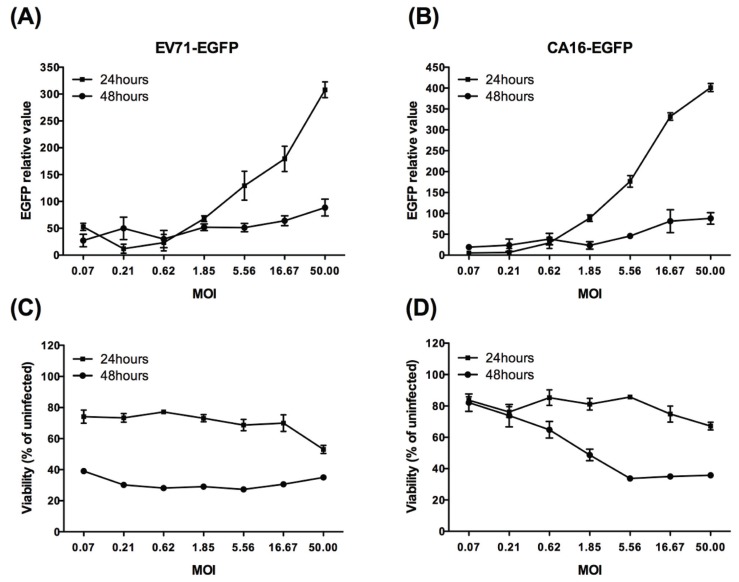
Correlation between EGFP intensity, virus-induced CPE and different multiplicity of infection (MOI) for EV71-EGFP (**A**, **C**) or CA16-EGFP (**B**, **D**). RDS cells in 96-well plates were infected with serially diluted EV71-EGFP or CA16-EGFP, and the plates were detected for EGFP signal (**A**, **B**) and cell viability (**C**, **D**) at 24 and 48 hpi. The relative EGFP expression was calculated by subtracting the background fluorescence intensity in uninfected cells from that in infected cells. Data are presented as the mean ± SD of three independent experiments.

To develop EGFP reporter viruses for an EGFP-based screening assay, appropriate conditions for infection were determined initially. RDS cells were infected with EGFP reporter virus at different MOIs in duplicate 96-well plates. One of the plates was used to read the fluorescence signal, while the other was applied in an MTT assay to detect the EGFP reporter virus-induced CPE. As shown in Figure 2A,B, a positive correlation existed between the EGFP signal and MOI at 24 h post‑infection (hpi), but the fluorescence signal was barely detected at 48 hpi. On the other hand, the MTT results (Figure 2C,D) demonstrated that most cells were alive at 24 hpi, while most cells had died at 48 hpi. Therefore, the MOI of 5.56 for EV71-EGFP and CA16-EGFP at 24 hpi, which resulted in a sufficient fluorescence signal with minimal CPE, was chosen for the antiviral screening assay.

We have recently described a high-throughput neutralization assay developed by utilizing non‑proliferating EV71 and CA16 pseudoviruses [29,30]. In this study, we employed these pseudoviruses to establish a luciferase-based antiviral screening assay by modifying the neutralization assay.

After the establishment of reporter virus-based screening assays, a primary screen was performed using a library of 400 highly purified natural compounds. The Z’ factor was monitored in each plate [38]. The fluorescence or luminescence values were normalized using the following equation: T − Cc/Vc − Cc, where T is the signal of compound-treated cells, Vc is the signal of virus control, and Cc is the signal of cell control. A total of 44 compounds that strongly reduced EGFP and/or luciferase expression (fluorescence and/or luminescence value < 0.5) were identified as positive hits (Supplementary Figure S1). The Z’ factors of screening plates were in the range of 0.5 to 0.7, which indicate a high quality screening system [38].

#### 3.2.2. Secondary Screening Using Cell Viability-Based Methods

To eliminate false positive and cytotoxic compounds, these 44 hits were re-evaluated by cell viability-based secondary screening. The correlation between MOI and cell viability was tested (data not shown), and an appropriate MOI of 0.005 was selected for the 48-h infection, which sufficiently resulted in nearly complete cell destruction (cell viability < 10%). 

**Figure 3:**
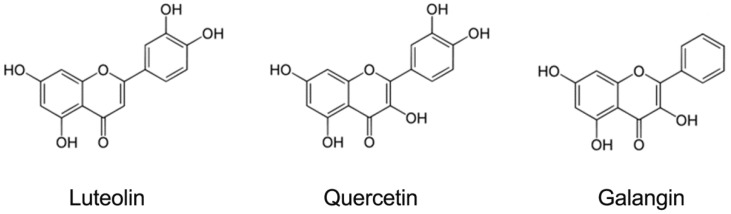
Chemical structures of identified flavonoids.

After the two-step screening, three primary candidates (luteolin, galangin, and quercetin) were identified from 400 natural compounds. Interestingly, these three compounds share similar structural features (Figure 3). Among them, luteolin showed the most potent inhibitory effect on EV71 and CA16 infection, and was chosen for further evaluation. 

### 3.3. Evaluation of Antiviral Activity of Luteolin against EV71 and CA16 Infection

**Figure 4:**
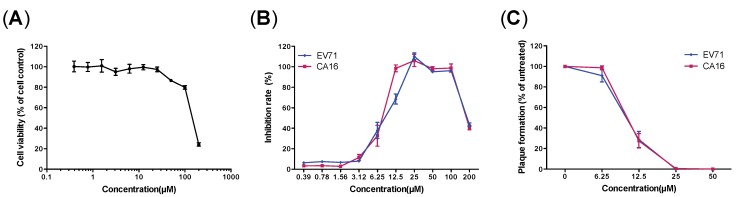
Cytotoxic effects and anti-EV71/CA16 activity of luteolin. (**A**) CC_50_ was determined by comparison of relative luciferase luminescence of luteolin-treated cells with that of untreated wells (dimethyl sulfoxide (DMSO) only) after incubation for 48 h. (**B**) Antiviral EC_50_ values for luteolin were determined by comparison of viabilities at 48 h after infection with EV71/CA16 (normalized and untreated cells). (**C**) Effect of luteolin on EV71/CA16 plaque formation. Data arepresented as the ratio of plaque numbers in the luteolin treated group to that in the untreated control. All results are represented as the mean ± SD of three independent experiments. CC_50_ and EC_50_s were calculated using linear regression analysis (Table 1).

To evaluate the antiviral activity and cytotoxicity of luteolin, RDS cells were treated with two-fold dilutions of the compound in the concentration range of 0.39–200 μM, with or without viral infection. The inhibitory effect on CPE was detected using the same assay described in the secondary screening. As indicated in Figure 4B, luteolin showed an EC_50_ of 10.307 μM for EV71 and 7.391 μM for CA16 and a CC_50_ of 148.02 μM (Figure 4A), resulting in a 50% selectivity index (SI_50_) of 14.36 for EV71 and 20.03 for CA16. The peak antiviral activity of the compound was achieved within the range from 25 to 100 μM. Furthermore, the antiviral activity and cytotoxicity of luteolin was evaluated in RD and Vero cells using the same methods to determine the EC_50_ and CC_50_ values (Table 1).

Next, the activity of luteolin was confirmed via plaque reduction tests. As indicated in Figure 4C, the amount of plaques markedly decreased with the increase of luteolin concentration. The estimated EC_50_ values of luteolin were 10.37 μM for EV71 and 10.52 μM for CA16. These results suggest that luteolin can effectively inhibit CPE induced by EV71 and CA16 infection at non-cytotoxic concentrations.

**Table 1:** Biological activity, cytotoxicity, and SI_50_ of luteolin in different cell lines.

Virus	CC_50_ (μM) ^a^			EC_50_ (μM) ^a^			SI_50_ (μM) ^b^		
	RDS	RD	Vero	RDS	RD	Vero	RDS	RD	Vero
EV71	148.02	292.00	178.65	10.31	31.56	25.11	14.36	9.25	7.11
CA16	148.02	292.00	178.65	7.39	14.87	7.16	20.03	19.64	24.95

^a^ CC_50_ and EC_50_ were determined with the CellTiter-Glo® Luminescent Cell Viability method. ^b^ SI_50_ is the ratio of CC_50_ to EC_50_.

**Figure 5:**
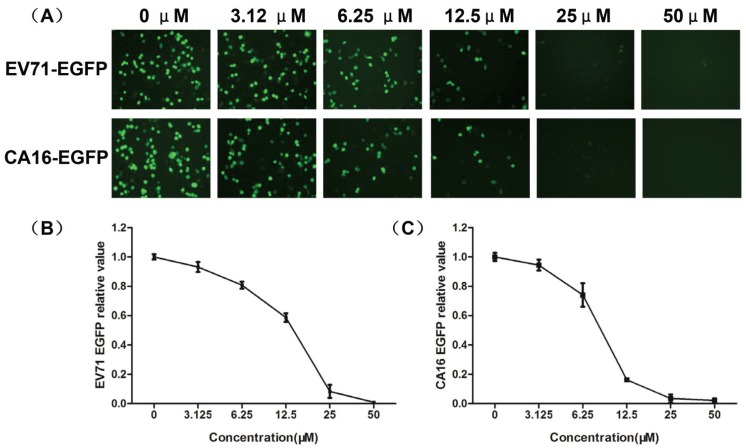
Antiviral activity of luteolin by using EV71-EGFP or CA16-EGFP. Confluent RDS cells in black 96-well plates were infected with EV71-EGFP or CA16-EGFP virus at the MOI of 5.56 and incubated with various concentrations of luteolin (0–50 μM). EGFP expression was detected by fluorescent microscopy (**A**) and automated fluorometry (**B**, **C**) at 24 hpi. Three independent experiments were performed in duplicate, and representative data are shown.

As an additional confirmatory assay, the inhibitory activity of luteolin on EV71-EGFP or CA16‑EGFP infection was examined by both fluorescent microscopy and automated fluorometry. As shown in Figure 5, the number of EGFP-positive cells reduced with the increasing concentration of luteolin, consistent with the automated fluorometry results. The EC_50_ of luteolin was estimated to be 14.25 μM for EV71 and 8.65 μM for CA16, similar to the results of the CPE reduction assay. 

### 3.4. Luteolin Targets the Post-Attachment Stage of EV71 and CA16 Infection

**Figure 6:**
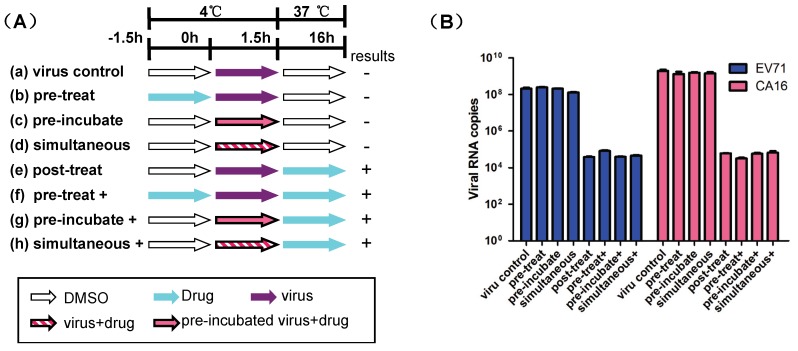
Time-of-addition assay. (**A**) RDS monolayers were inoculated with EV71/CA16 and luteolin at the indicated time periods and temperatures. Procedure (**a**) was the virus control. In procedure (**b**), the monolayers were pre-treated with luteolin before EV71/CA16 infection. Procedure (**c**) involved pre-incubation of luteolin with the virus prior to infection. In addition, the cells were treated with the compound and virus simultaneously in procedure (**d**) as a control of procedure (**c**). In procedure (**e**), the cells were first exposed to the virus before adding luteolin. Procedures (**f**), (**g**), and (**h**) were modified from procedures (**b**), (**c**), and (**d**) by also adding the test compound after the 1.5 h time point, which is represented by “+” in the graph. At the end of each time period (the arrow in the graph), the monolayers were washed twice to remove the unattached virus and/or excess luteolin. (**B**) Antiviral effects of luteolin under the procedures (**a**–**h**) were detected via real-time RT-PCR. The results are presented as the mean ± SD of three independent experiments.

To examine the potential inhibitory mechanisms, stages of the enterovirus replication cycle targeted by luteolin were determined by modification of a previously described assay [14]. As described in Figure 6A, eight different procedures were performed, and viral RNA levels were measured. In pre‑attachment inhibition procedures (b, c, and d), only negative results (similar to virus control) were observed (Figure 6B), while the post-attachment inhibition procedure (e) resulted in more than a 1000‑fold reduction of EV71 and CA16 RNA synthesis (Figure 6B). These results implied that luteolin targeted the post-attachment stage. To further confirm this result, we modified the pre‑attachment inhibition procedures (b, c, and d) by also adding the test compound after the 1.5 h time point (f, g, and h) (Figure 6A). As expected, results similar to procedure (e) were observed (Figure 6B). Together, these results imply that luteolin targets the post-attachment stage of EV71 and CA16 infection. 

**Figure 7:**
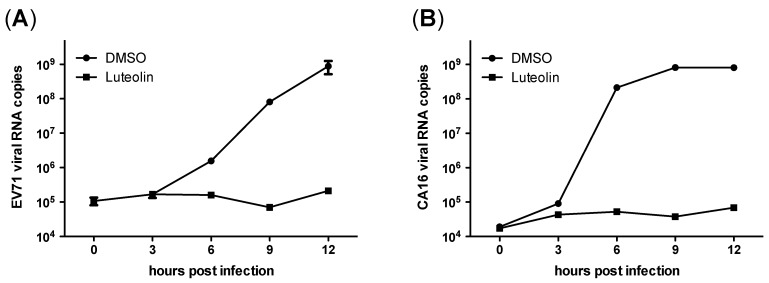
Effect of luteolin on viral RNA replication. Detection of viral RNA in cells treated with luteolin or DMSO after EV71 (**A**) or CA16 (**B**) infection. Each data point represents two determinations, and representative results from one of two separate experiments are shown.

To further investigate the inhibitory mechanism, we examined the kinetics of viral RNA replication. RDS cells were inoculated with EV71/CA16 and then washed and treated with luteolin (50 μM) or not (only DMSO). Viral RNA levels were detected at indicated time points. In the initial three hours, viral RNA increased similarly in treated and untreated cells (Figure 7). Subsequently, viral RNA increased dramatically in untreated cells, while they remained constant in luteolin-treated cells. The results indicated that luteolin could inhibit EV71 and CA16 replication, even if added at 4 hpi (data not shown).

## 4. Discussion

HFMD outbreaks are among the most pressing health concerns in the Western Pacific region, and effective antiviral therapies against EV71 and CA16 infections are urgently needed. In previous studies, a dozen synthetic compounds were found to be effective *in vitro* or *in vivo* [17,18,19,39]. Great efforts also have been made to identify effective compounds or components from herbs or traditional Chinese medicine. Some traditional Chinese herb extracts were found to inhibit cytopathic effects [40,41]. However, it is difficult to characterize the antiviral mechanisms of these whole extracts as they contain a mixture of different components. Recently, a number of natural compounds has been reported to have inhibitory activities against EV71/CA16 [15,16,42,43]. Since most of these compounds were isolated from herbs already commonly used for HFMD therapy, many natural resources remain to be further investigated.

Three types of approaches have been utilized to identify antiviral compounds. The first approach is mechanism based and detects the enzyme activity of a purified viral protein. This approach has been developed for screening inhibitors against enteroviruses by targeting polymerase 3D^pol^ [44] and protease 2A^pro^ [45] and 3C^pro^ [46]. The second approach is based on a reporter protein encoding the replicon or infectious virus, and the detected fluorescence or luminescence signal is considered to be in proportion to the virus replication. This approach can identify multiple targets of the virus growth cycle [47]. The third approach involves assessing cell viability by detecting the viral CPE. This method has been utilized to identify inhibitors for human rhinovirus and coxsackie B virus [35,48]. Nevertheless, a major problem with this assay is that it cannot discriminate between drug toxicity and viral CPE. Since the second and third approaches are based on cells, which involve the cellular uptake of test compounds, inhibitors identified from these assays have a higher success rate in following studies *in vivo*. Moreover, it should be noted that reporter virus-based assays only are suitable for identifying antiviral activity from non-toxic compounds, since toxic compounds would decrease the fluorescence or luminescence signal. Thus, the antiviral screening assay in this study was developed by combining the second and third approaches in order to avoid false negative or false positive results.

A small-scale screening was performed on a natural product library. Our screening system identified three compounds (luteolin, quercetin, and galangin) as positive hits. Among them, quercetin has recently been reported to inhibit the replication of EV71/CA16 [42]. Therefore, having quercetin independently selected as a hit further supported the reliability of our screening system. 

As the most promising hit, anti-EV71/CA16 activity of luteolin was further evaluated in a dose‑dependent manner. The EC_50_ values were measured using two ‘gold standard’ methods (CPE and plaque reduction assay) and an EGFP reporter virus-based assay. Similar EC_50_ values were obtained from these three assays, reflecting the sensitivity and reliability of the EGFP-based antiviral assay. The SI of luteolin was calculated as 14.36 for EV71 and 20.03 for CA16. Prior to validation of this important criterion it was determined that the SI value of ≥4 should be considered suitable for an antiviral agent [49]. Furthermore, the anti-EV71/CA16 activity of luteolin was reproduced when tested in both RD and Vero cells, two cell lines that can be infected by EV71/CA16, suggesting that such an inhibitory effect is cell-type independent. Luteolin and other two flavones were also reported to inhibit the infection of poliovirus, which is another enterovirus. The EC_50_ of luteolin in anti-poliovirus experiments was 40 μM, presenting comparable activity with this anti-EV71/CA16 assay [50].

Analysis of the targeted stage of infection was preformed to investigate the inhibitory mechanism of luteolin. The results verified that luteolin did not effect EV71/CA16 binding and entry (Figure 6). In addition, real-time RT-PCR detection of viral RNA (Figure 7) confirmed that luteolin functioned after attachment and blocked the viral RNA synthesis. Several studies have been published on the antiviral activity of luteolin. For instance, Mehla *et al.* [51] reported that luteolin cripples HIV-1 Tat function at the level of Tat-LTR transactivation (transcriptional step). Meanwhile Manvar *et al.* [52] reported that luteolin inhibits RNA polymerase activity of HCV. These findings indicate that further studies should focus on RNA-dependent RNA polymerase (RdRp) assays. 

Luteolin, quercetin, and galangin, which are structurally related natural flavones, are believed to possess the ideal chemical structure as anti-oxidants and free-radical scavengers [53]. Their chemical structures differ by only one to three hydroxyl groups. In addition, two o-methylated flavonols, chrysosplenetin and penduletin, have recently been reported to have strong activity against EV71 *in vitro*. These observations strongly suggest that flavonoids have potential anti-EV71/CA16 activities, although the mechanisms remain to be elucidated, and further assessments of the relationships between structure and activity are needed.

In conclusion, this *in vitro* study validates a very useful approach to developing antiviral agents against EV71/CA16 using a reporter virus and a cell viability-based antiviral screening system. The potent inhibitor luteolin, identified from a highly purified natural product library, may be a promising candidate for antiviral therapy as well as a useful research tool for EV71/CA16 infections.

## Supplementary Files

Supplementary File 1 Supplementary Materials (PDF, 390 KB)
