# Supplementary material for: Identification of Luteolin as Enterovirus 71 and Coxsackievirus A16 Inhibitors through Reporter Viruses and Cell Viability-Based Screening
Source: Viruses. 2014 Jul 17;6(7):2778–95. doi: 10.3390/v6072778 (PMC4113793; doi:10.3390/v6072778)

Supplementary Materials

Identification of Luteolin as Enterovirus 71 and Coxsackievirus A16 Inhibitors through Reporter Viruses and Cell Viability-Based Screening

Lin Xu, Weiheng Su, Jun Jin, Jiawen Chen, Xiaojun Li, Xuyuan Zhang, Meiyan Sun, Shiyang Sun, Peihu Fan, Dong An, Huafei Zhang, Xiguang Zhang, Wei Kong, Tonghui Ma and Chunlai Jiang

Figure S1. Antiviral activity of hits from the reporter virus-based primary screen. The fluorescence or luminescence values were normalized using the following equation: T − Cc/Vc − Cc, where T is the signal of compound-treated cells, Vc is the signal of virus control, and Cc is the signal of cell control. A total of 44 hits, which produced normalized fluorescence or luminescence values below 0.5, showed more than 50% inhibition of EGFP and/or luciferase reporter virus infection. All compounds were tested at a final concentration of 100 μM.

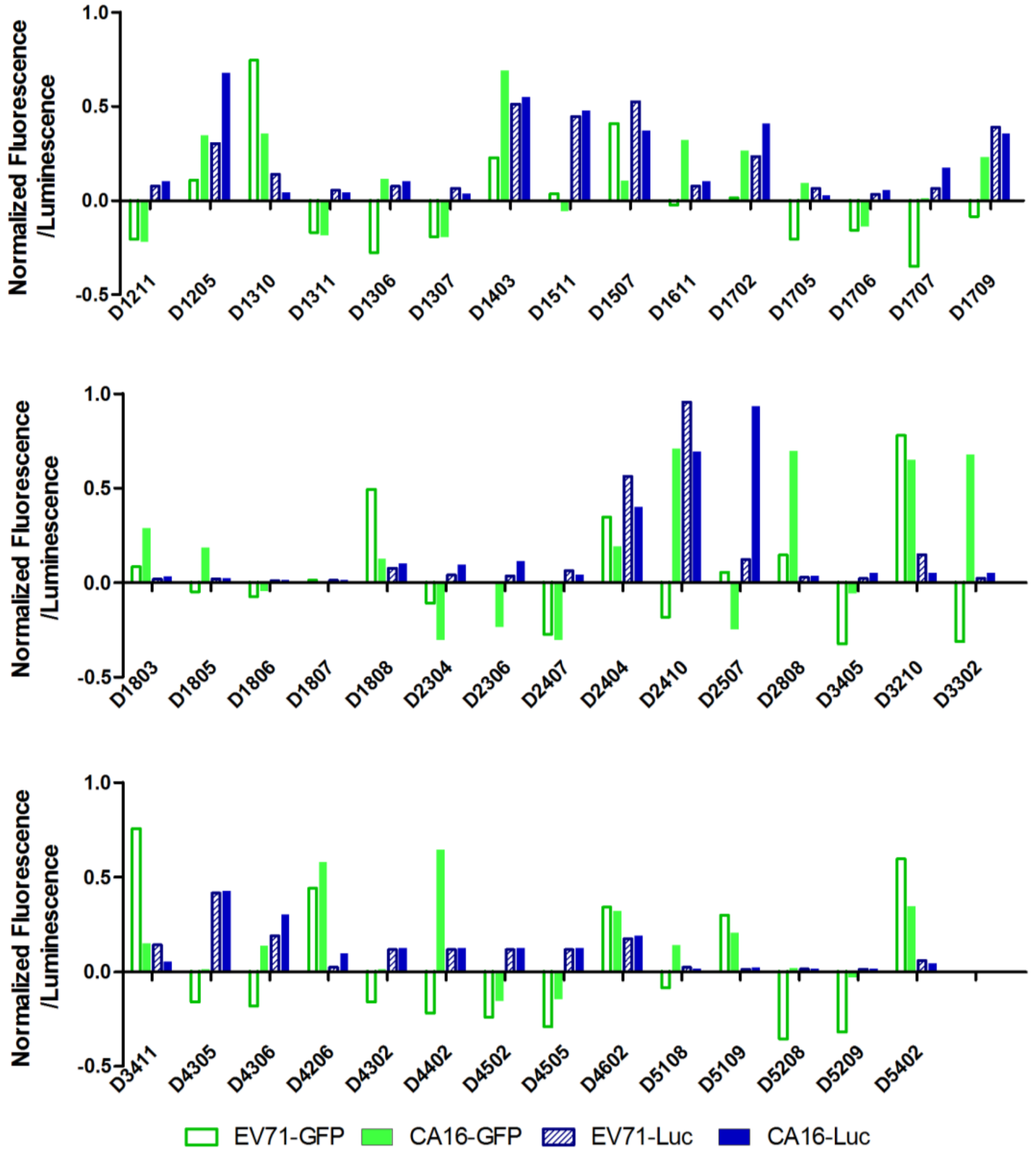

Supplement: Supplementary File 1 — Supplementary Materials (PDF, 390 KB) [file viruses-06-02778-s001.pdf]
